# The Mode of Pretransplant Dialysis Does Not Affect Postrenal Transplant Outcomes in African Americans

**DOI:** 10.1155/2012/303596

**Published:** 2012-09-12

**Authors:** Amit Sharma, Todd L. Teigeler, Martha Behnke, Adrian Cotterell, Robert Fisher, Anne King, Todd Gehr, Marc Posner

**Affiliations:** ^1^Department of Surgery, Hume-Lee Transplant Center, Virginia Commonwealth University, Richmond, VA 23298, USA; ^2^Hume-Lee Transplant Center, Virginia Commonwealth University Health Systems, P.O. Box 980057, Richmond, VA 23298, USA

## Abstract

*Background*. In previous reports with a majority of Caucasian patients, peritoneal dialysis (PD) before kidney transplantation has been associated with poor outcomes and higher rates of graft thrombosis and infectious complications than hemodialysis (HD). We report our experience on the outcomes of prerenal transplant peritoneal dialysis in predominantly (73%) African American patient population. *Methods*. A retrospective data analysis of 401 kidney transplants performed at our center from 2000 to 2006 was performed. Adult recipients with at least three months of pretransplant HD or PD were included. *Results*. There were 339 patients on HD and 62 patients on PD. There was no difference in graft (*P* = 0.51) and patient survival (*P* = 0.52) at 1, 3, and 5-years. Patients on HD were more likely to experience delayed graft function than PD (38.8% versus 17.7%, *P* < 0.005). There was no difference in the incidence of vascular thrombosis or posttransplant infectious complications. When only the African American patients in the two groups were compared, there were no differences in graft or patient survival. *Conclusions*. Pretransplant peritoneal dialysis is associated with excellent patient and renal allograft outcomes in African Americans and does not predispose them to an increased risk of infectious or thrombotic complications.

## 1. Introduction

Renal transplantation remains the treatment of choice for many patients with end-stage renal disease (ESRD). However, the effect of dialysis modality on posttransplant outcomes has been the subject of longstanding debate. While peritoneal dialysis (PD) has been reported to favorably influence early graft function after renal transplantation compared to hemodialysis (HD) [1, 2], one registry analysis was associated with poor early renal allograft survival in patients on PD [3].

ESRD patients on PD have been reported to be more likely to have allograft vascular thrombosis compared to patients treated with HD [4–7]. The effect of pretransplant dialysis modality on early posttransplant infections also remains controversial. While some studies have noted a higher incidence of infections in patients on PD in the first month after transplant [8], others have found no difference in the rate of posttransplant infections in patients receiving PD [9, 10]. Others have reported a higher rate of posttransplant infections in patients on HD [11].

A common denominator in most studies that have compared pretransplant PD to HD has been a predominance of Caucasian patients. More than 60% of ESRD patients at our center are African Americans and an increasing percentage is being referred for peritoneal dialysis. To our knowledge, there are no single center studies that have compared outcomes of pretransplant PD to HD in African American patients with ESRD. The primary goal of this single-center, retrospective analysis was to compare patient and graft survival between prerenal transplant peritoneal and hemodialysis patients in a predominantly African American population with ESRD. We also studied the incidence of postoperative complications with focus on posttransplant vascular thrombosis, infectious complications, and delayed graft function.

## 2. Materials and Methods

### 2.1. Study Design

A retrospective data analysis of all renal transplants performed at our institute between 2000 and 2006 was done. The dialysis modality at the time of renal transplantation was recorded. Adult recipients with at least three months of pretransplant HD or PD were included in this analysis. Medical records were reviewed along with divisional electronic database to determine patient and graft survival and the incidence of vascular, infectious, and immunological complications after transplantation. Recipients with retransplants, with multiple-organ transplants, positive for HIV, or receiving kidneys from donors less than 18 years old were excluded from the study. The study protocol was approved by the Institutional Review Board.

### 2.2. Renal Transplant and Followup

Renal transplants from living or cadaveric donors were performed as per standard surgical practice. In patients with PD, the peritoneal dialysis catheter was removed at the time of transplant and patients with delayed graft function were managed with hemodialysis using temporary access. All transplant recipients received antibody induction using rabbit anti-thymocyte globulin (rATG; Thymoglobulin; Genzyme Corp, Cambridge, MA) from day 0 to day 3 posttransplant (1.5 mg/kg). Maintenance immunosuppression consisted of cyclosporine/tacrolimus, mycophenolate mofetil, and tapering doses of steroids for life.

Renal allograft vascular anatomy was evaluated on postoperative day 1 by color duplex Doppler sonography. After hospital discharge, renal graft function was monitored by measuring the serum creatinine levels. *Graft rejection* was detected on histopathology of ultrasound-guided percutaneous biopsy performed after an unexplained rise in serum creatinine levels of >25%. *Graft loss* was defined as patient requiring removal of the renal allograft, permanent return to dialysis, retransplantation, or recipient death. *Delayed graft function* (DGF) was defined as need for dialysis in the first week after transplant. The incidence of systemic infections requiring antimicrobial therapy during first year was noted along with local wound infections at the renal transplant incision. The incidence of acute rejections and vascular complications, especially arterial or venous thrombosis, in the first year after transplant was studied.

### 2.3. Statistical Analysis

Differences between groups were analyzed with Chi-square test for categorical variables and by independent *t*-tests for continuous variables. Univariate patient and graft survival were analyzed with the Kaplan-Meier estimator, and differences in patient and graft survival were determined by Mantel-Cox log rank test using SPSS version 19 (SPSS Inc., Chicago, IL, USA). Multivariate Cox proportional hazards survival analysis was performed on variables achieving univariate significance at the *P* value < 0.05, using SAS v9.3 (SAS Institute, Inc., Cary, NC).

## 3. Results

Of the 659 kidney transplants performed at our center between January, 2000 and December, 2006, 401 recipients met the inclusion criteria outlined above. The dialysis modality was HD in 339 and PD in 62 recipients. Seventy-three percent of recipients were African Americans (75% in HD and 63% in PD group).

The demographics of kidney donors and recipients are summarized in Table 1. A higher proportion of the donors for the HD group were African American (39.5% versus 25.8%, *P* = 0.046). The recipients in the PD group were younger (44 ± 13.8 versus 47.6 ± 12.7, *P* = 0.04) and a lower proportion were males (38.7% versus 56%, *P* = 0.01) than those in the HD group. The recipient groups were comparable in proportion of African Americans, duration of pretransplant dialysis, pretransplant panel reactive antibody levels (PRA), HLA mismatch, cold ischemia time (CIT), and warm ischemia time (WIT). There were no differences in the etiology of ESRD, recipient serological status for cytomegalovirus, or immunosuppression protocols used (data not shown).

**Figure 1:**
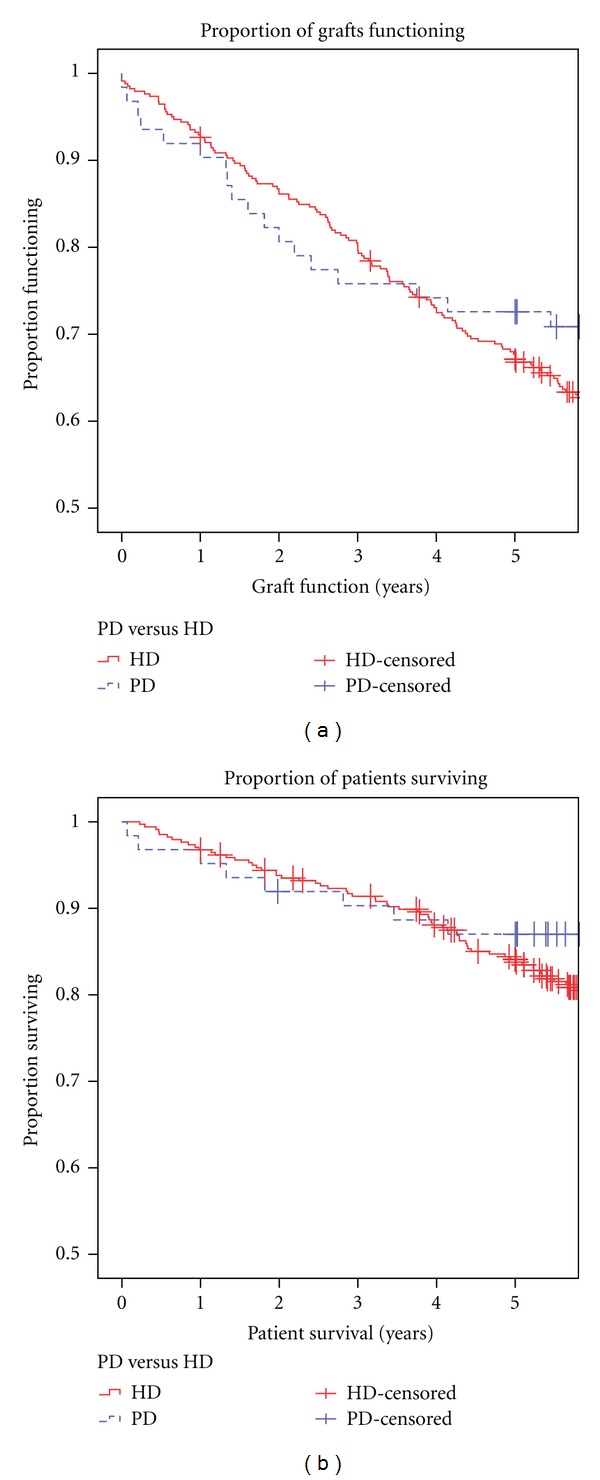
Graft (a) and patient survival (b) curves in peritoneal (PD) and hemodialysis (HD) groups. By Mantel-Cox log rank test, there was no difference in graft (*P* = 0.51) or patient (*P* = 0.52) survival.

**Figure 2:**
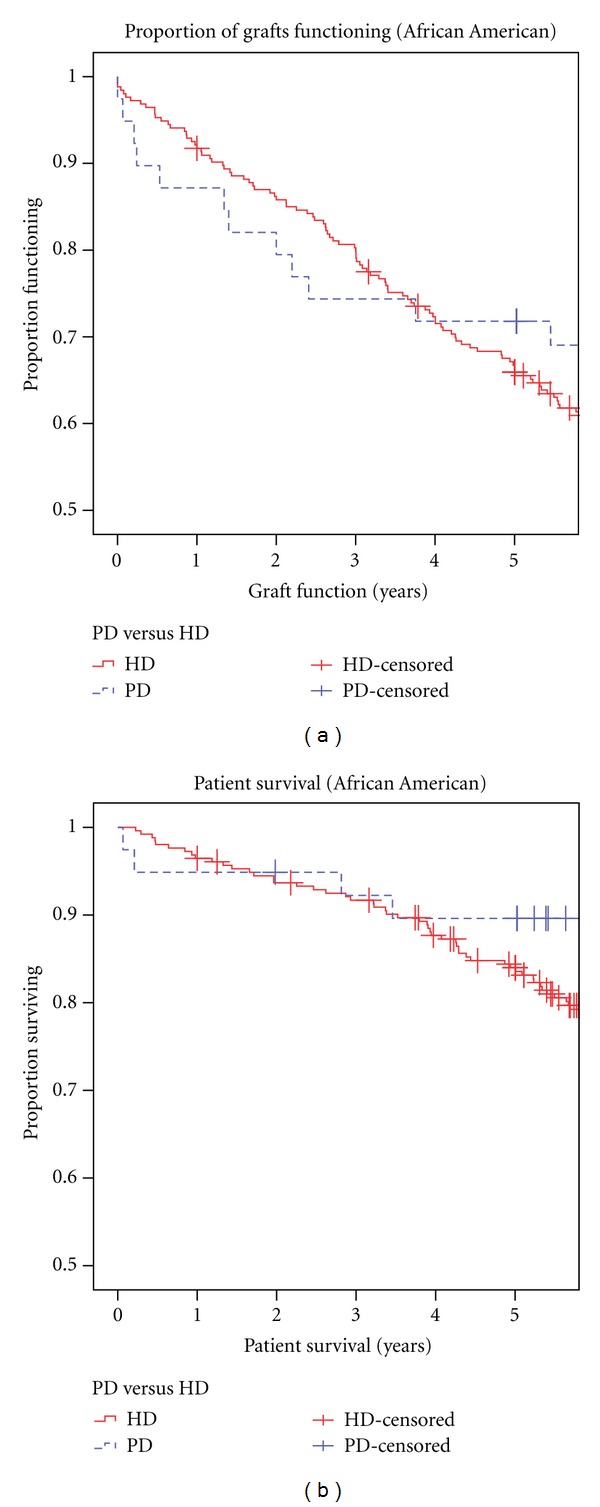
Graft (a) and patient (b) survival curves in African American subgroup with pretransplant peritoneal (PD) and hemodialysis (HD). By Mantel-Cox log rank test, there was no difference in graft (*P* = 0.46) or patient (*P* = 0.3) survival.

There were no significant differences in the 1-, 3-, and 5-year graft or patient survival between the two groups (Table 2). Patient and graft survival curves are shown in Figure 1. Mantel-Cox log rank test showed no difference in graft (*P* = 0.51) or patient (*P* = 0.52) survival (Figure 1). Patient and graft survival for the African American sub-group that received prerenal transplant PD versus HD, were not significantly different by Mantel-Cox log rank test (Figure 2). Multivariate Cox proportional hazards survival analysis showed that age and sex were not significant predictors of patient or graft outcomes. Neither race nor duration of time on dialysis influenced outcomes in the PD versus HD subanalysis (*P* = 0.73 and 0.88, resp.). In the multivariate analysis, DGF but not dialysis modality was a significant variable for patient and graft survival (*P* = 0.002).

The complications during the first year after renal transplant are shown in Table 2. The incidence of DGF was significantly lower in the PD group compared to the HD group (17.7% versus 38.8%, *P* < 0.005). We did not find any significant difference in the incidence of arterial or venous thrombosis, systemic or local wound infections, or acute rejections episodes between the two groups.

## 4. Discussion

To our knowledge, this is the first single-center study to compare the outcomes of prerenal transplant peritoneal to hemodialysis in a predominantly African American population. While most reports from the United States had only 20–30% African American patients [3, 6, 12, 13], 73% of our renal allograft recipients were African Americans. We have shown that long-term graft survival is independent of the modality and duration of dialysis. Patients on pretransplant PD had a significantly lower rate of DGF, and there was no increased risk of graft vascular thrombosis or infectious complications.

Since PD has been shown to be more cost-effective and reduce rehospitalization rates [14] in addition to preserving residual renal function [15], there is a renewed interest to promote this dialysis modality for ESRD patients in United States. While some studies have failed to find a difference in outcomes [12, 16–18], others have found PD to have beneficial effects after renal transplantation compared to HD [1, 13, 19]. Our findings are similar to the studies [3, 13, 19] that have shown PD to have a protective effect on lowering the rate of DGF. In our study, the HD and PD groups were well matched for most donor and recipient characteristics with 75% and 63% African American patients, respectively. Patient survival was similar in the two groups. In the multivariate analysis, DGF was the only factor with a significant impact on graft survival. Due to the relatively small sample size of this study, age and sex matching the two comparison groups would be underpowered. Therefore, the multivariate Cox proportional hazards survival analysis was used to detect the influence of differences in demographic characteristics on outcomes and it showed that age and sex were not significant predictors of patient or graft outcomes. While some studies [12, 20] have shown the length of time on dialysis to affect transplant outcomes, we did not find any such association in this analysis. Like others [12, 16, 18], we observed that the dialysis modality and race did not independently affect graft outcomes.

End-stage renal disease patients on PD have been reported to be more likely to have allograft vascular thrombosis compared to patients treated with HD [4–7]. The incidence of vascular thrombosis at our center was very low with only two cases in the HD group. None of the patients in the PD group had any graft vascular thrombosis. This overall low rate could be attributed to the short cold ischemia times in our study for both HD and PD groups (10.1 ± 9.7 and 12.4 ± 11.0 hours, resp.) as others have associated longer cold ischemia time with increased risk of vascular thrombosis especially in pediatric kidney transplant recipients [21]. Unfortunately, it would not have been appropriate to calculate a *P* value for differences in graft thrombosis between HD and PD groups in our sample, but one can be optimistic that the point estimate for graft thrombosis after pretransplant PD does not represent a higher risk to our patients.

A higher rate of sepsis has been reported in PD compared to HD patients elsewhere [22] which has been attributed to microbial seeding of the peritoneal cavity in the pretransplant period. In our study, there was no significant difference between the two groups in the incidence of systemic or local wound infections during the first year after renal transplantation. While some studies have reported a risk of acute cellular rejection in patients receiving PD [8], we did not find any evidence to support their claim.

The main drawback of this study is its retrospective nature. There were fewer patients in the PD group as we were interested in a minimum five-year posttransplant follow-up. The strengths of this study are the use of uniform surgical and immunosuppressive protocols in well-matched PD and HD groups.

We conclude that dialysis modality is not a predictor of long-term graft outcomes after renal transplantation. Pretransplant peritoneal dialysis is associated with good long-term renal allograft survival in African American patients. PD is associated with lower rates of delayed graft function and does not predispose to renal allograft vascular thrombosis, infections, or acute rejections. Transplant nephrologists and surgeons should not sacrifice the possible economic, lifestyle, and psychological benefits of peritoneal dialysis based on unfounded fears of poor renal transplant outcomes.

## Figures and Tables

**Table 1:** Donor and recipient demographics.

	Hemodialysis (*n* = 339)	Peritoneal dialysis (*n* = 62)	*P* value
Kidney donor			
Age (yr)	39.8 ± 14.7	41.2 ± 16.9	ns
Males	167 (49.3%)	38 (61.3%)	ns
African American	134 (39.5%)	16 (25.8%)	*P* = 0.046
SCD	210 (61.9%)	41 (66.1%)	ns
ECD	23 (6.8%)	4 (4.5%)	ns
DCD	8 (2.4%)	1 (1.6%)	ns
Living donors	98 (28.9%)	16 (25.8%)	ns
Kidney recipient			
Age (yr)	47.6 ± 12.7	44 ± 13.8	*P* = 0.04
Males	190 (56%)	24 (38.7%)	*P* = 0.01
African American	254 (74.9%)	39 (62.9%)	ns
Time on dialysis (mo)	44.0 ± 39.2	35.2 ± 32.8	ns
PRA	26.9 ± 36.3	23.7 ± 34.8	ns
HLA mismatch	4 ± 1.6	4 ± 1.7	ns
CIT (hr)	10.1 ± 9.7	12.4 ± 11.0	ns
WIT (min)	30.6 ± 8.8	31.7 ± 11.7	ns

SCD: standard criteria donors, ECD: extended criteria donors, DCD: donation after cardiac death, PRA: panel reactive antibodies, HLA: human leukocyte antigen, CIT: cold ischemia time, WIT: warm ischemia time, ns: not significant (*P* > 0.05).

**Table 2:** Dialysis modality and renal transplant outcomes.

	Hemodialysis (*n* = 339)	Peritoneal dialysis (*n* = 62)	*P* value
Patient survival (%)			
1 year	96.8 ± 1.0	95.2 ± 2.7	ns
3 years	91.4 ± 1.5	90.3 ± 3.8	ns
5 years	84.1 ± 2.0	87.0 ± 4.3	ns
Graft survival (%)			
1 year	92.6 ± 1.4	90.6 ± 3.8	ns
3 years	80.2 ± 2.2	75.8 ± 5.4	ns
5 years	67.1 ± 2.6	72.6 ± 5.7	ns
Outcomes (1st year)			
DGF	130 (38.8%)	11 (17.7%)	*P* < 0.005
Vascular thrombosis	2	0	
Systemic infections	99 (29.2%)	15 (24.2%)	*P* = 0.45
Wound infections	20 (5.9%)	2 (3.2%)	*P* = 0.36
Rejection	49 (14.5%)	4 (6.5%)	*P* = 0.10
Length of stay (d)	6.6 ± 3.3	6.6 ± 2.5	*P* = 0.99

Patient and graft survivals are given as percentage ± standard error, DGF: delayed graft function, ns: not significant (*P* > 0.05).
